# Not All Fevers Equal Infection: A Challenging Case of Adult-Onset Still's Disease

**DOI:** 10.7759/cureus.59968

**Published:** 2024-05-09

**Authors:** Nkechi Ukoha, Kikelomo Olaosebikan, Patrick Berchie, Fawwad A Ansari, Zola Nlandu

**Affiliations:** 1 Internal Medicine, Piedmont Athens Regional Medical Center, Athens, USA; 2 Infectious Disease, Piedmont Athens Regional Medical Center, Athens, USA

**Keywords:** adult-onset still's disease (aosd), anakinra, disease-modifying antirheumatic drugs (dmards), hyperferritinemia, yamaguchi, immune-mediated inflammatory disorder, corticosteroids

## Abstract

Adult-onset Still's disease (AOSD) stands as a perplexing condition with diverse clinical manifestations, posing significant diagnostic challenges for healthcare professionals. This case report delves into the clinical trajectory, diagnostic challenges, treatment strategies, and outcomes experienced by a 67-year-old female with AOSD. This report advocates for considering AOSD as a potential diagnosis in patients presenting with systemic inflammatory symptoms, especially when other conditions have been ruled out. It highlights the complexity of AOSD and the importance of interdisciplinary collaboration, close monitoring, and personalized treatment strategies to optimize patient outcomes.

## Introduction

Adult-onset Still's disease (AOSD) is a puzzling and complicated condition. While primarily afflicting adults, its rarity and wide-ranging clinical manifestations frequently present healthcare professionals with significant diagnostic hurdles [1]. To illuminate the intricate path AOSD traverses, this case report delves into the compelling clinical trajectory, the treatment strategies employed, and ultimately the outcome experienced by a patient grappling with this disorder.

In the last two decades, we have seen a large body of immunological research on cytokines, which has attributed to both a better understanding of AOSD and significant advances in treatment [2]. Through a thorough analysis of our patient's journey, including diagnostic dilemmas, therapeutic decisions, and clinical responses, this report aspires to shed light on the nuanced manifestations and complexities inherent to AOSD. By doing so, we hope to stimulate further research, encourage collaborative learning, and ultimately improve patient outcomes by refining diagnostic approaches and tailoring treatment regimens to address the unique challenges posed by AOSD.

## Case presentation

A 67-year-old female without significant past medical history and in a monogamous relationship presented to the hospital for the evaluation of worsening polyarthralgia, myalgia, fever, sore throat, and fleeting rash. Her symptoms started suddenly about a week before the presentation, and the patient visited the emergency room where she was evaluated for syphilis. Rapid plasma reagin (RPR) titer was 1:2. She was started on intramuscular penicillin and a short course of low-dose oral prednisone for latent syphilis infection and was discharged home to follow up with infectious disease as an outpatient. 

**Figure 1:**
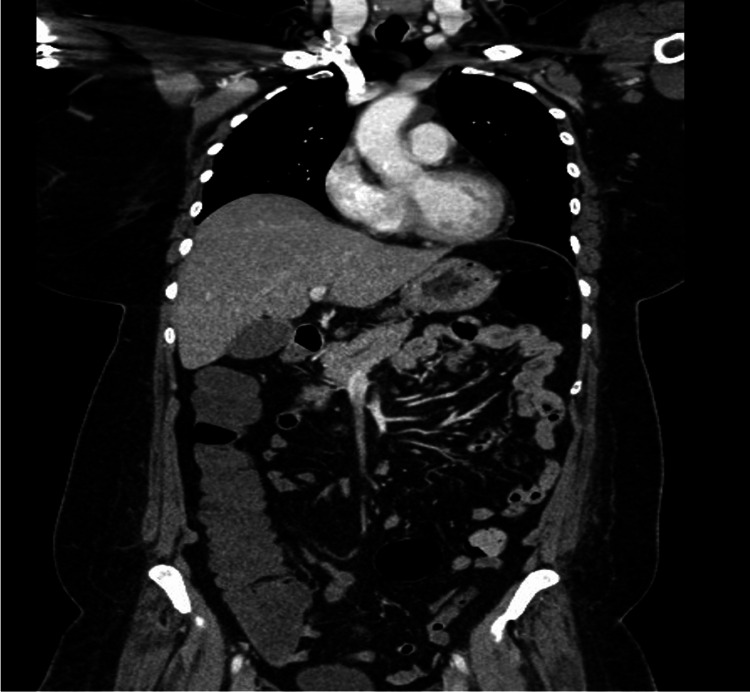
Computed tomography of the chest, abdomen, and pelvis, with contrast in coronal plane No evidence of infection or acute abnormalities was observed in the chest, abdomen, and pelvis

The patient presented again a day after discharge due to unchanged symptoms. On arrival, she was afebrile, tachycardic, and tachypneic with a blood pressure of 94/66 mmHg. Pertinent labs include leukocytosis of 42.2x10^3^/µL, C-reactive protein (CRP) >16 mg/dl, a ferritin level of over 7500 ng/mL, hemoglobin 11.5 g/dl, aspartate transaminase 79 U/L, alanine transaminase 107 U/L, alkaline phosphatase 200 U/L, and total bilirubin 0.8 mg/dl and non-reactive hepatitis panel. The chest radiograph was unremarkable. She was admitted with a working diagnosis of sepsis without a clear source and started on empiric antibiotics (piperacillin-tazobactam and vancomycin). A multidisciplinary team was involved in conjunction with us the infectious disease team. Broad-spectrum antibiotics were subsequently discontinued when no definitive infection was found as evidenced by unremarkable blood and urine culture, human immunodeficiency virus (HIV), fungal studies, viral studies, and imaging studies (see Figure 1).

A repeat RPR and fluorescent antibody absorption at our facility was unreactive, and her outpatient RPR was thought to be a false positive. She was diagnosed as having a fever of unknown origin with differentials of AOSD versus hemophagocytic lymphohistiocytosis (HLH). Hematology was consulted, and workup for HLH, including bone marrow biopsy with flow cytometry, was unrevealing. However, with additional history-taking and a review of all available clinical data, the patient met the Yamaguchi criteria for AOSD with quotient fever, arthralgia, evanescent rash, leukocytosis, sore throat, and abnormal liver enzymes supporting its diagnosis. Her anti-nuclear antibody (ANA) was positive with a titer of 1:80 and a homogeneous pattern which is nonspecific and can be seen in juvenile idiopathic arthritis, and her rheumatoid factor was negative. The patient was started on high-dose steroids with improvement in symptoms and a downward trend of laboratory markers and was thereafter transferred to another facility for rheumatological review. After review by a rheumatologist, she was continued on high-dose steroids with methylprednisolone 1 gram daily given intravenously with marked improvement of symptoms. She was discharged on prednisone 60 mg daily in addition to anakinra 100 mg/0.67 mL subcutaneously twice a day, to follow up as an outpatient.

## Discussion

This process was first described in children by George Still, "Still's disease," in 1896. In 1971, Eric Bywaters further characterized the term to include "adult Still's disease" which was used to describe a series of adult patients with similar symptoms as seen in children [1]. The etiology of AOSD is unknown, and both genetic factors and a variety of infectious triggers have been proposed; however, the evidence for both is scarce [3]. It is a very uncommon disease with an estimated incidence of 0.16-0.4 per 100,000 adults. It has a bimodal age distribution that peaks between the ages of 15-25 years and 36-45 years [4]. 

The hallmark of AOSD involves neutrophil and macrophage activation triggered by the pro-inflammatory cytokine IL-18. Neutrophil (PMN) CD64, a neutrophil activation marker, has been found to be upregulated in active AOSD [1,5]. The condition has been associated with markedly elevated ferritin, in the absence of infection [6]. The presence of symptoms and laboratory abnormalities based on the Yamaguchi criteria comprise major criteria (fever >39°C (102.2°F) for >1 week, arthralgia/ arthritis >2 weeks, typical rash which is non-pruritic macular and salmon-colored, and >10,000 WBCs per microliter with >80% polymorphonuclear cells) and minor criteria (sore throat, lymphadenopathy, abnormal liver function test, negative ANA, negative rheumatoid factor, and hepatomegaly or splenomegaly). To make the diagnosis based on the Yamaguchi criteria, the presence of five features, with at least two being major, is diagnostic, and it is important to exclude infections, malignancy, and rheumatological diseases [7]. 

This case highlights the diagnostic challenges encountered in identifying AOSD due to its overlapping symptoms with other systemic inflammatory conditions. The patient initially presented with polyarthralgia, myalgia, fever, and a fleeting rash, which in conjunction with an initial RPR titer of 1:2 led to a suspected diagnosis of syphilis. However, despite receiving treatment for possible latent syphilis infection, the patient's symptoms persisted, prompting further investigation. Infectious disease workup, including extensive laboratory testing and imaging, did not identify an infectious etiology for the patient's symptoms. Due to continued symptoms, the possibility of AOSD or HLH was considered. An unremarkable bone marrow biopsy and meeting the Yamaguchi criteria supported the diagnosis of AOSD.

Our patient's diagnostic journey underscores the importance of thorough clinical evaluation and exclusion of alternative diagnoses. A tentative diagnosis of AOSD was made based on the patient meeting the Yamaguchi criteria, which include fever, arthralgia or arthritis, typical rash, leukocytosis, and negative tests for other rheumatic diseases.

Managing AOSD necessitates a comprehensive, multidisciplinary strategy encompassing diagnosis, symptom management, and long-term complication prevention. While NSAIDs have limited efficacy in achieving remission for most AOSD patients, short-term use may be considered during diagnostic workups or early relapses [8]. High-dose corticosteroids constitute the primary treatment for AOSD across clinical presentations, proving more effective in addressing systemic symptoms. However, prolonged corticosteroid use carries potential side effects including gastrointestinal bleeding, hypertension, diabetes, cataracts, osteoporosis, psychosis, and weight gain, urging caution [7].

Should corticosteroid treatment fail, or dependency develop, disease-modifying antirheumatic drugs (DMARDs) may be explored [8]. While certain DMARDs, such as cyclosporine A, leflunomide, azathioprine, hydroxychloroquine, and tacrolimus, have demonstrated some efficacy based on retrospective cases, their general applicability remains limited, reserved for severe complications and instances where other specific treatments falter. Methotrexate, on the other hand, stands as a favorable and primary steroid-sparing treatment for AOSD [9-11].

However, our patient upon transfer to an outside facility was administered anakinra, a non-glycosylated variant of the human IL-1 receptor antagonist (IL-1Ra). Anakinra's mechanism involves binding to IL-1 receptors (IL-1RI), obstructing their activation by IL-1α or IL-1β [7]. Notably, anakinra marked a significant milestone as the first biological agent to exhibit efficacy in treating both systemic and articular AOSD symptoms [12]. Clinical trials have indicated that its effectiveness is heightened when administered early in the disease course, particularly benefiting those with highly active systemic AOSD [7]. 

## Conclusions

AOSD remains a challenging condition with a myriad of clinical presentations and diagnostic difficulties. Through this case report, our aim was to emphasize the importance of heightened awareness, timely diagnosis, and a multidisciplinary approach to managing this rare inflammatory disorder effectively. Further research is warranted to elucidate the underlying mechanisms and develop even better-targeted therapies for improved patient care and outcomes.
